# Hydroxypropyl-β-cyclodextrin as an effective carrier of curcumin – piperine nutraceutical system with improved enzyme inhibition properties

**DOI:** 10.1080/14756366.2020.1801670

**Published:** 2020-09-23

**Authors:** Anna Stasiłowicz, Ewa Tykarska, Kornelia Lewandowska, Maciej Kozak, Andrzej Miklaszewski, Joanna Kobus-Cisowska, Daria Szymanowska, Tomasz Plech, Jacek Jenczyk, Judyta Cielecka-Piontek

**Affiliations:** a Department of Pharmacognosy, Faculty of Pharmacy, Poznan University of Medical Sciences, Poznań, Poland; b Department of Chemical Technology of Drugs, Poznan University of Medical Sciences, Poznan, Poland; c Department of Molecular Crystals Institute, Molecular Physics Polish Academy Sciences, Poznan, Poland; d Department of Macromolecular Physics, Adam Mickiewicz University in Poznan, Poznan, Poland; e Division of Functional Nanomaterials, Poznan University of Technology, Poznan, Poland; f Department of Gastronomy Sciences and Functional Foods, Faculty of Food Science and Nutrition, Poznan University of Life Sciences, Poznan, Poland; g Department of Biotechnology and Food Microbiology, Poznan University of Life Sciences, Poznan, Poland; h Department of Pharmacology, Medical University of Lublin, Lublin, Poland; i NanoBioMedical Centre, Adam Mickiewicz University, Poznań, Poland

**Keywords:** Curcumin, piperine, cyclodextrin, hyaluronidase, acetylcholinesterase

## Abstract

The nutraceutical system of curcumin-piperine in 2-hydroxypropyl-β-cyclodextrin was prepared by using the kneading technique. Interactions between the components of the system were defined by X-ray powder diffraction (XRPD), differential scanning calorimetry (DSC), infrared spectroscopy (FT-IR), nuclear magnetic resonance (NMR). Application of hydroxypropyl-β-cyclodextrin as a carrier-solubiliser improved solubility of the curcumin–piperine system, its permeability through biological membranes (gastrointestinal tract, blood–brain barrier) as well as the antioxidant, antimicrobial and enzyme inhibitory activities against acetylcholinesterase and butyrylcholinesterase.

## Graphical Abstract

**Figure:**
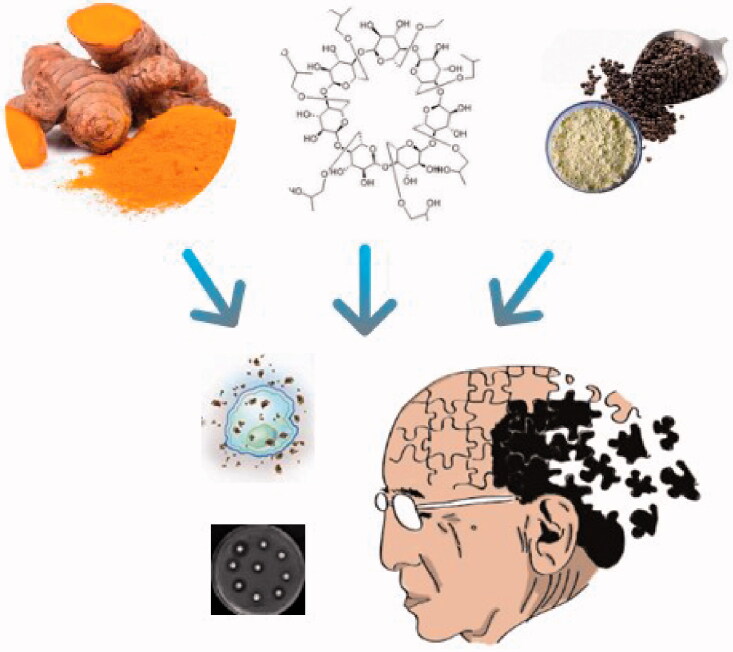

## 1. Introduction

Curcumin is a polyphenol compound that originates from the rhizome of the herb *Curcuma longa*. The turmeric, family *Zingiberaceae* has been known to humanity for centuries as a spice, dye, and natural remedy for many health problems. Considering the statistics on turmeric sales in the USA published in 2015 by Statista, along with forecasts for the coming years, there is a growing trend of interest in this plant. The *Curcuma longa* rhizome contains curcuminoids, among them about 77% curcumin, 17% demethoxycurcumin, and 6% bisdemethoxycurcumin. Diferuloylmethane, called curcumin, is a bis-α,β-unsaturated β-diketone, which exhibits keto-enol tautomerism. Regardless of the tautomeric form, curcumin exhibits multidirectional therapeutic properties, such as antioxidant, anti-inflammatory, antimicrobial, and neuroprotective effects. These properties were confirmed during studies on various animal models and several clinical trials^1–4^. Unfortunately, the medical use of curcumin is limited for many reasons: i) low solubility – curcumin is practically insoluble in water, ii) poor absorption, iii) high metabolism rate, iv) rapid elimination from the body. Various approaches were reported to overcome the limitations of using curcumin in the prevention or treatment of many disorders. These studies are all the more justified because toxicity to high doses of curcumin has not been proven^5^. The best tested solutions to increase the bioavailability of curcumin included: i) increasing its solubility by combining with selected solubilises (e.g. cyclodextrin derivatives)^6^, ii) using adjuvants (e.g. with piperine)^7^, iii) preparation of nanoparticles^8^, iv) formulation of liposomes^9^, micelles^10^ and phospholipid complexes containing synthetic drug associated with curcumin (e.g. oxaliplatin and curcumin complexes)^11^, v) synthesis of derivates and analogues with better bioavailability and different metabolic ways^12^. Solubility modification and/or combination with metabolism modifiers, e.g. epicatechin or piperine, are the simplest solutions that can also be used in the development of pharmaceutical dosage forms, dietary supplements, as well as in the application of functional food.

In the literature of the subject, there are reports on the combination of curcumin with solubilisers from the cyclodextrin group. C. S. Mangolim et al.^13^, proved that curcumin introduced into the cavity of β-cyclodextrin by co-precipitation method had 30-fold enhanced solubility, and was less susceptible to degradation under the influence of sunlight, pH, temperature. Another study conducted by V. R. Yadav et al.^14^ showed that curcumin seems to be the best included in hydroxypropyl-β-cyclodextrin among all-natural cyclodextrins and the complex is a promising solution in the treatment of inflammatory bowel disease due to its better anti-inflammatory and anticancer properties. A recent study by A. Celebioglu et al.^15^ reported that it was possible to obtain an orally fast-dissolving food supplement containing curcumin and hydroxypropyl-β-cyclodextrin by using the electrospinning technique.

A combination of curcumin with metabolism modifiers can contribute to increasing its clinical activity. Curcumin has been shown to enlarge its bioavailability in combination with piperine – an alkaloid that occurs in black pepper, long pepper, and other plants of the *Piperaceae* family. Piperine is a known inhibitor of hepatic and intestinal glucuronidation. Both substances were orally administered to humans and rats in order to study the effect of piperine on curcumin bioavailability. The results of increasing the bioavailability of curcumin by the alkaloid were much more noticeable in humans than in the rat model. The test also showed that the administration of such a combination has no negative impact on either animals or humans, but has proven that piperine increased the absorption and bioavailability of curcuminoids^7^.

It should be noted that the application of piperine in free form is also limited due to its low water solubility, low bioavailability, and high pungency^16^. Therefore, a few current studies reported the benefits of incorporating piperine into cyclodextrins. M. Quilaqueo et al.^17^ proved that the inclusion of piperine in β-cyclodextrin improves its permeability as the result of the modification of an active transport mechanism, especially for the β-cyclodextrin:piperine system (4:1). The study by T. Ezawa et al.^18^ confirmed that solubility of piperine after its incorporation into the cavity of β-cyclodextrin increased about 16-folds.

In order to prove the benefits of combining curcumin and piperine with cyclodextrins to increase their solubility, permeability, and biological activity, the nutraceutical system of curcumin – piperine incorporated into 2-hydroxypropyl-β-cyclodextrin was designed.

Our studies involved: (i) the preparation and identification of nutraceutical system of curcumin – piperine with the 2-hydroxypropyl-β-cyclodextrin as carrier, (ii) studies on changes in solubility and permeability through artificial biological membranes simulating gastrointestinal wall and blood–brain barrier, (iii) evaluation of the biological activity of ternary nutraceutical system of curcumin – piperine (antioxidant potential, hyaluronidase, acetylcholinesterase, and butyrylcholinesterase inhibition and antimicrobial activity).

## 2. Experimental part

### 2.1. Raw materials and reagents

The raw plant material of turmeric (assay ≥65% of curcuminoids), piperine with a purity >97%, and 2-hydroxypropyl-β-cyclodextrin (molar substitution 0.8) were purchased from Sigma-Aldrich (Poznan, Poland). The reference standard of curcumin (purity >99.5%) was also supplied from Sigma-Aldrich (Poznan, Poland). Acetonitrile (HPLC grade) was provided by Merck KGaA (Darmstadt, Germany), and other chemical reagents: acetic acid, methanol (HPLC grade), dimethyl sulfoxide (HPLC grade), sodium chloride and potassium dihydrogen phosphate were obtained from Avantor Performance Materials (Poland). Prisma HT, GIT Lipid solution, Acceptor Sink Buffer were supplied by Pion Inc (UK).

### 2.2. Identification of turmeric extract

In order to establish the content of the main ingredient – curcumin in turmeric extract, high-performance liquid chromatography with DAD detector (HPLC-DAD) was used. As a reference standard – curcumin was applied with the purity >99,5%. The separation of curcumin and other curcuminoids (demethoxycurcumin and bisdemethoxycurcumin) was achieved under the following conditions: a stationary phase Phenomenex-C18 column (250 mm × 4,6 mm; 5 µm), the column temperature – 303 K, a mobile phase comprised 1% acetic acid and acetonitrile (45:55, v/v), the flow rate was 1.0 mL/min, the injection volume was 20 µL. The DAD detection wavelength was set at 421 nm.

### 2.3. Preparation of curcumin – piperine – 2-hydroxypropyl-β-cyclodextrin nutraceutical system

Kneading technique was used to prepare the system of curcumin – piperine – 2-hydroxypropyl-β-cyclodextrin. The molar ratio of curcumin and piperine was 1:1. In the nutraceutical system the mass ratio of curcumin:hydroxypropyl-β-cyclodextrin was 1:1, and of piperine:hydroxypropyl-β-cyclodextrin was also 1:1. In the first stage, curcumin and piperine were ground in the mortar. Next, all three substances with a minor amount of alcohol and distilled water to receive a pasty consistency were ground for 60 min. The system was drying in 45 °C for 24 h, and later it was ground in mortar again for 10 min. The physical mixture of compounds in the same ratio was also prepared.

### 2.4. Identification of curcumin – piperine – 2-hydroxypropyl-β-cyclodextrin nutraceutical system

Interactions between curcumin, piperine, and 2-hydroxypropyl-β-cyclodextrin (HP-β-CD) in solid-state were examined using X-ray powder diffraction (XRPD), differential scanning calorimetry (DSC), infrared spectroscopy (FT-IR), solid state nuclear magnetic resonance (NMR).

#### 2.4.1. X-ray powder diffraction (XRPD)

Analysis of the complexation process was performed by X-ray powder diffraction (XRPD) method. Diffraction patterns were recorded on a PANalitycal Empyrean diffractometer with CuK_α_ radiation (1.54056 Å) at a tube voltage of 45 kV and a tube current of 40 mA. The angular range was 3° to 50° with a step size of 0.017° and counting rate of 15 s/step. OriginPro 8 was used to analyse the acquired data^19^.

#### 2.4.2. Differential scanning calorimetry

Thermal analysis was carried out using DSC 204 Phoenix differential scanning calorimeter. The powdered samples of 5 mg were placed in hermetically enclosed aluminium cells next to the reference sample (air) and heated at a scanning rate of 5 K min^−1^ from 294 to 568 K in a helium atmosphere with a flow rate of 40 mL min^−1^. The details of the experimental procedure and data processing were identical to those described by us for other pharmaceutical compounds^20–23^.

#### 2.4.3. Infrared spectroscopy and DFT calculations

Infrared spectra were performed on an FT-IR Bruker Equinox 55 spectrometer. The substances, the system and physical mixture were turned into pellets with IR grade KBr in the hydraulic press, and then the spectra were recorded in a frequency range of 400–4000 cm^−1^. The DFT calculations with a B3LYP functional and 6–31 G(d,p) as basis set were used as supportive methods.

#### 2.4.4. Solid state NMR analysis

NMR ^13^C spectra were recorded on a 400 MHz Agilent spectrometer equipped with Wide Bore Triple Resonance T3 MAS XY probe. Cross-Polarisation (CP) pulse sequence with Magic Angle Spinning (MAS) and dipolar decoupling of protons was applied. Samples were placed into a 4 mm diameter zirconia rotor and spun with 7 kHz.

### 2.5. Apparent solubility and permeability through membranes simulating gastrointestinal walls and brain–blood barrier of the curcumin – piperine – 2-hydroxypropyl-β-cyclodextrin nutraceutical system

Differences in concentrations of curcumin and piperine during solubility study were measured by high-performance liquid chromatography with the DAD detector (HPLC-DAD). Conditions for the determination of curcumin by HPLC-DAD method are given in section 2.2. The piperine determination was carried out using a Phenomenex-C18 column (250 mm × 4.6 mm; 5 µm) as the stationary phase and a mixture of methanol and water (80:20, v/v) as the mobile phase. The column temperature was set at 303 K. The flow rate of the mobile phase was 1.4 mL/min, while the detection wavelength was set at 343 nm. The analysis was performed with the injection volume set to 100.0 µL.

The changes in concentrations of curcumin and piperine during permeability studies were recorded spectrophotometrically. The determinations of curcumin were conducted at 421 nm and piperine at 343 nm.

The dissolution study was performed in the paddle apparatus. Curcumin, piperine, and their system with cyclodextrin were weighed to gelatine capsules, which were later implemented to springs to sink and prevent flotation on the surface of the medium. The study was carried out at a pH of 6.8. The vessels were filled with 500 mL of phosphate buffer, the temperature was maintained at 310 K, the paddles were set at the stirring speed of 100 rotations per minute. The 5.0 mL samples were withdrawn at predetermined time points with the replacement of equal volumes of temperature-equilibrated media and filtered through a membrane filter (0.45 μm). The dissolution profiles were compared with the use of two-factor values *f*_1_ and *f*_2_^24^ implemented by Moore and Flanner^25^ according to the following equations:

$$f_{1}=\frac{\sum_{j=1}^{n}\left|R_{j}-T_{j}\right|}{\sum_{j=1}^{n}R_{j}}\times 100$$

$$f_{2}=50\;\times \log\left({\left(1+(\frac{1}{n})\sum_{j=1}^{n}{\left|R_{j}-T_{j}\right|}^{2}\right)}^{-\frac{1}{2}}\times 100\right)$$

where *n* is the number of time points, *R_j_* is the percentage of the reference dissolved substance in the medium, *T_j_* is the percentage of the dissolved tested substance, *t* is the time point. Dissolution profiles are described as similar when the *f*_1_ value is close to 0, or *f*_2_ is close to 100 (between 50 and 100)^26^.

*In vitro* gastrointestinal (GIT) and blood–brain barrier (BBB) permeability was studied using the PAMPA model (Parallel Artificial Membrane Permeability Assay). The sandwich consists of two 96-well microfilter plates. The PAMPA system contains two chambers: the donor at the bottom and the acceptor chamber at the top. The chambers are separated by a 120-μm-thick microfilter disc coated with a 20% (w/v) dodecane solution of a lecithin mixture (Pion, Inc.). Before adding samples to the donor compartments, the samples were dissolved in DMSO. The donor solution was adjusted to pH ≈ 6.8 for GIT application and to pH ≈ 7.4 for BBB application using 0.5 M NaOH. The plates were combined and then incubated for 3 h for GIT model and 4 h for BBB model in a humidity-saturated atmosphere at the temperature set at 310 K. The apparent permeability coefficient (*P_app_*) was calculated according to the following equation:

$$P_{\mathrm{app}}=\frac{-ln(1-\frac{C_{A}}{C_{\mathrm{equilibrium}}})}{S\times (\frac{1}{V_{D}}+\frac{1}{V_{A}})\times t}$$

where *VD* – donor volume, *VA* – acceptor volume, *Cequilibrium* – equilibrium concentration $C_{\mathrm{equilibrium}}=\frac{C_{D}\times V_{D}+\;C_{A}\times V_{A}}{V_{D}+V_{A}},$ *S* – membrane area, *t* – incubation time (in seconds). Compounds with the value of *P_app_* in GIT model below 0.1 × 10^−6 ^cm s^−1^ are described as low permeable, substances found as medium permeable have a 0.1 × 10^−6 ^cm s^−1^ ≤ *P_app_* < 1 × 10^−6 ^cm s^−1^ and compounds with a *P_app_* ≥ 1 × 10^−6 ^cm s^−1^ are defined as ones with high permeability^27^.

Substances whose *P_app_* in BBB model is <2.0 × 10^−6 ^cm s^−1^ are defined as low permeable. API with the *P_app_* value in the range of 2.0–4.0 × 10^−6 ^cm s^−1^ are described as substances with questionable permeability. Compounds with high permeability have the *P_app_* value at the level >4.0 × 10^−6 ^cm s^−1^ ^28^.

### 2.6. Biological activity of the curcumin – piperine – 2-hydroxypropyl-β-cyclodextrin nutraceutical system

#### 2.6.1. Antioxidant activity

The antioxidant activity was evaluated using the DPPH radical reaction. The investigation of antioxidant properties was performed spectrophotometrically, according to Studzińska-Sroka’s modifications^29^. Methanol solution of DPPH (0.2 mM) and ascending concentrations of curcumin, piperine alone and in the system with 2-hydroxypropyl-β-cyclodextrin were prepared. 25.0 µL of DPPH solution was mixed with 175.0 µL of studied solutions in a 96-well plate, and then the plate was shaken and incubated in darkness for half an hour at room temperature. Subsequently, the absorbance (A) was measured at the wavelength set at 517 nm against the blank (the mixture of DPPH solution and methanol). The inhibition of the DPPH radical by the studied samples was calculated due to the following equation:

$$A=\frac{A_{o\;}-\;A_{i}}{A_{o}}\;x\;100\%$$

where *A*_o_ is the absorbance of the control sample, *A_i_* is the absorbance of studied samples. Every determination was repeated six times. As a reference, vitamin C was used. IC_50_ values, which determine the concentration of the substance that inhibits the formation of DPPH by 50%, were determined by linear regression analysis.

#### 2.6.2. Determination of hyaluronidase inhibition

The inhibition of hyaluronidase was investigated using the turbidimetric method’s Grabowska with slight changes^30^. The study was performed in the 96-well filter plate. To the plate was added 25.0 µL of incubation buffer, 25.0 µL of hyaluronidase solution, 10.0 µL of the sample, 15.0 µL of acetate buffer (pH 4.5). Afterward, the plate was kept for 15 min in the temperature of 310 K. Hyaluronic acid (25.0 µL) was then added to the cells, and another incubation was carried out for 45 min at 310 K. Finally, 200.0 µL of hexadecyltrimethylammonium bromide (CTAB) solution was added, and cells were incubated for 10 min at room temperature (293 K). The spectrophotometer with the wavelength set at 600 nm was used to measure the turbidance. Each test was repeated five times. The anti-hyaluronidase activity was calculated from the following equation:

$$I\%=\frac{\left(P-B_{3})-(B_{2}-B_{1}\right)}{\left(B_{4}-B_{3})-(B_{2}-B_{1}\right)}\;\times \;100\%$$

where: *P* – sample turbidance, *B*_1_ – absorbance of blank 1 (blind control), *B*_2_ – absorbance of blank 2 (with enzyme and hyaluronic acid – determination of enzyme properties), *B*_3_ – absorbance of blank 3 (investigating tested substances’ and system’s absorbance at 600 nm with the enzyme), *B*_4_ – absorbance of blank 4 – (investigating tested substances’ and system’s absorbance at 600 nm with the hyaluronic acid)

#### 2.6.3. Determination of acetylcholinesterase (AChE) and butyrylcholinesterase (BChE) inhibition

The modified spectrometric method developed by Ellman et al.^31^, which was described by Kobus-Cisowska at al.^32^ was used to measure the activity of curcumin, piperine, and their system with cyclodextrin. A POLARstar Omega (BMG LABTECH, Germany) plate reader was used for measurements of 96-well plates of the maximum volume of 300 μL. The hydrolysis of acetylthiocholine/butyrylthiocholine caused a colour change. The absorbance of the enzymes was measured at a wavelength of 412 nm, ten minutes after pipetting on a microplate. The reaction mixture containing 0.1 mL of 0.3 mM 5,5-dithio-bis-(2-nitrobenzoic acid) (DTNB, Sigma Aldrich, Germany), 10 mM NaCl and 2 mM MgCl2·6H2O solution, 0.575 mL 50 mM Tris-HCl buffer (pH = 8.0), 25 µL of 0.28 units/mL AChE/BChE (Sigma Aldrich, Germany) and 0.2 mL of tested extract was measured at wavelength 405 nm and at temperature 22 °C. The measurement was conducted after 20 min (BChE) or 60 min (AChE) after adding all ingredients into a microplate. The blank sample contained Tris-HCl buffer instead of tested compounds. A positive-negative control was applied and it consisted of 90.7 µM eserine instead of tested compounds. All samples were analysed in eight independent replicates. The inhibitory activity of each enzyme was calculated with the use of a calibration curve. The calibration curves were prepared using serine as a standard at concentration ranges between 0.09–6.10 µM for AChE and 0.09–8.57 µM for BChE.

#### 2.6.3. Determination of antimicrobial activity

The agar well diffusion technique was used to determine the antimicrobial activity of piperine, curcumin, and their system with cyclodextrin. Three 5 mm × 5 mm wells were made on the surface of the agar plate using a template. About 0.1 mL of solutions were delivered to the first well using a micropipette. 5.25% of sodium hypochlorite and 0.9% normal saline as positive and negative controls filled the other two wells. The samples were then incubated at 310 K for 24 h. Afterward, the development of clear zones around solutions was monitored. The antimicrobial activity was assessed by the diameter of the clear zone (inhibition zone).

## 3. Results and discussion

More and more knowledge about the synergism of the connections of known biologically active compounds promotes the development of multifunctional delivery systems. When designing ternary systems, we can plan to increase the strength of biological action as a result of combining two active compounds, but also to increase the strength of their biological action as a result of improving physicochemical parameters.

Bearing in mind the knowledge about the interactions of curcumin and piperine *in vivo* expressed as blocking the metabolic deactivation of curcumin by piperine, we combined these two components into a nutraceutical delivery system. We assessed the changes in the biological properties of curcumin and piperine as a result of preparation of the nutraceutical delivery system with cyclodextrin. The same physicochemical limitation of curcumin and piperine – low solubility – was eliminated by the design of the nutraceutical delivery system involving 2-hydroxypropyl-β-cyclodextrin as a carrier with solubilising properties. Similar approaches have already been reported for different ternary systems, where solubility, stability and potency were modified. For example Vieira, A. C. et al.^33^ described an increase of solubility and stability for efavirenz according to ternary system formation containing methyl-β-cyclodextrin and polyvinylpyrrolidone K-30. Wang, D. et al.^34^ noted the enlargement of the solubility and stability of dihydroartemisinin after preparing the ternary system with hydroxypropyl-*β*-cyclodextrin and lecithin. Ammar, H. O. et al.^35^ described the increase of dissolution efficiency of glimepiride in ternary systems with water-soluble polymers (PEG 4000) and cyclodextrins.

The nutraceutical system of curcumin and piperine with cyclodextrin was prepared using kneading technique. All preparation stages were repeatable, and incorporation processes were spontaneous as a result of mechanical action on system components. The overall process for obtaining the nutraceutical curcumin – piperine – 2-hydroxypropyl-β-cyclodextrin system was short, cheap and low-waste. The identity of the resulting system was tested using the following analytical techniques: XRPD, DSC, FT-IR and NMR.

**Figure 1.**
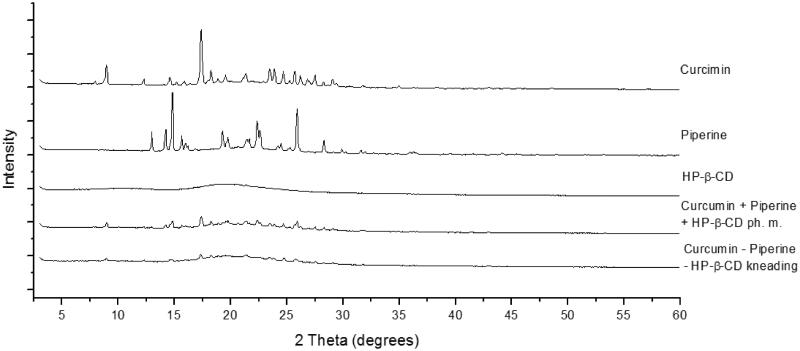
XRPD diffraction patterns of curcumin, piperine, their physical mixture (ph. m.) and system with hydroxypropyl-β-cyclodextrin (HP-β-CD) obtained by kneading method.

X-ray powder diffraction (XRPD) patterns of the tested samples are shown in Figure 1. Well-defined peaks on piperine and curcumin patterns indicate that these compounds exist in highly crystalline forms. The pattern of 2-hydroxypropyl-β-cyclodextrin is characteristic of an amorphous state. Diffractograms of the physical mixture and co-kneaded sample are aggregates of patterns of the individual components. Analysis of XRPD data indicates that the kneading method does not change the piperine and curcumin crystalline phases, and therefore does not influence their physicochemical properties. Thus, possible differences in the solubility of active compounds can only result from the use of cyclodextrin as a carrier.

**Figure 2.**
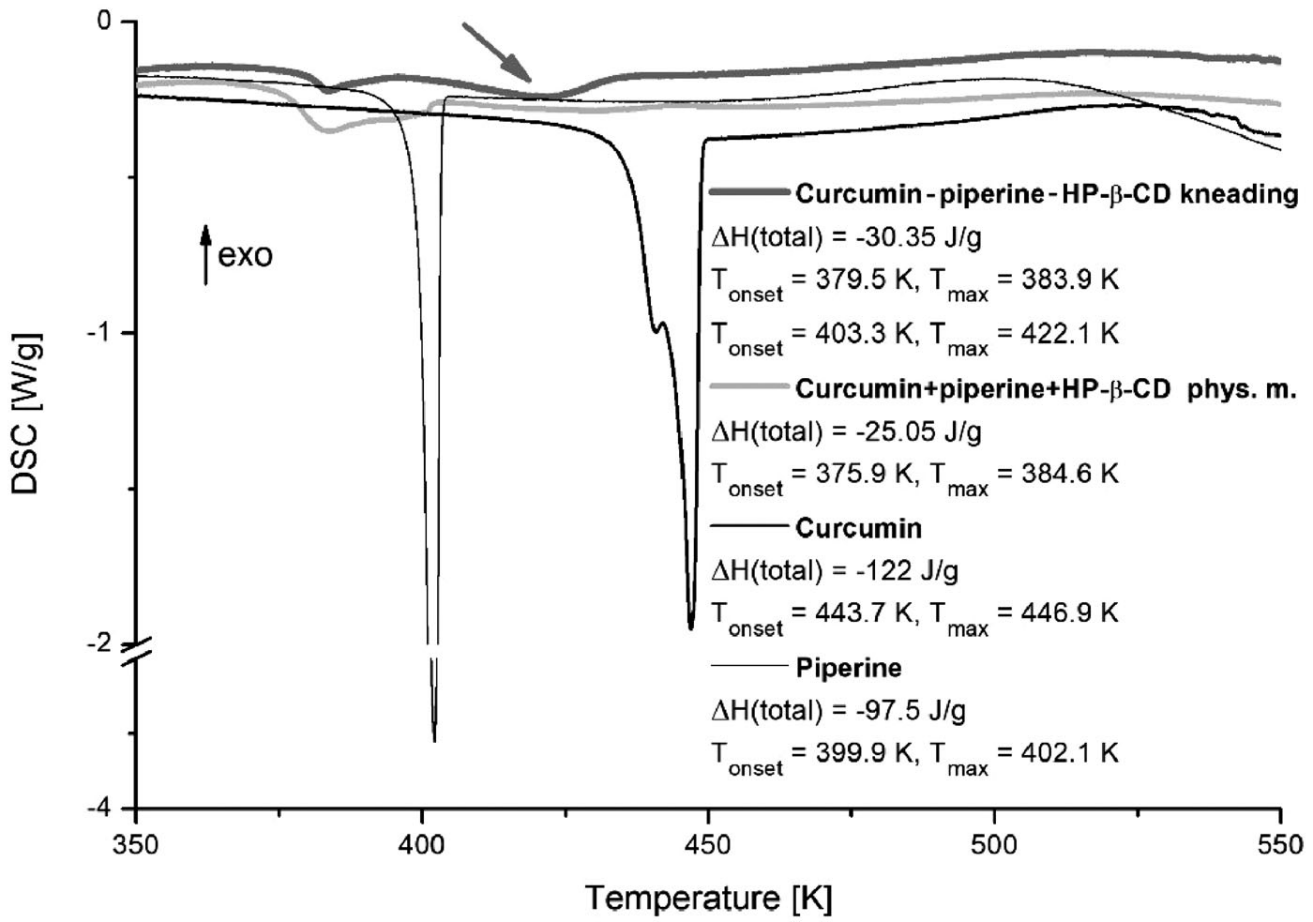
DSC thermograms of curcumin, piperine, their physical mixture, and system with hydroxypropyl-β-cyclodextrin (HP-β-CD) obtained by kneading method.

Thermodynamic parameters of tested substances were characterised using the DSC technique. Thermograms obtained for studied piperine, curcumin, their system with hydroxypropyl-β-cyclodextrin obtained by kneading method and physical mixture, presented in Figure 2, revealed a distinct difference in line-shapes. The main broad and asymmetric peak between 375 and 410 K (T_max_ = 384.5 K) is observed for the physical mixture. We can assume that the peak broadening is associated with the overlap of the peak ascribed to the melting of the free piperine phase (T_max_ = 402.1 K). For the system obtained by the kneading method we can observe a new peak (marked by the grey arrow in Figure 2) located between 403 and 440 K (T_max_ = 422.1 K). There is also the difference in total enthalpy values characterising phase transitions in both ternary combinations, where ΔH = −25.05 J/g and ΔH = −30.35 J/g, for physical mixture and system obtained by the kneading method, respectively.

**Figure 3.**
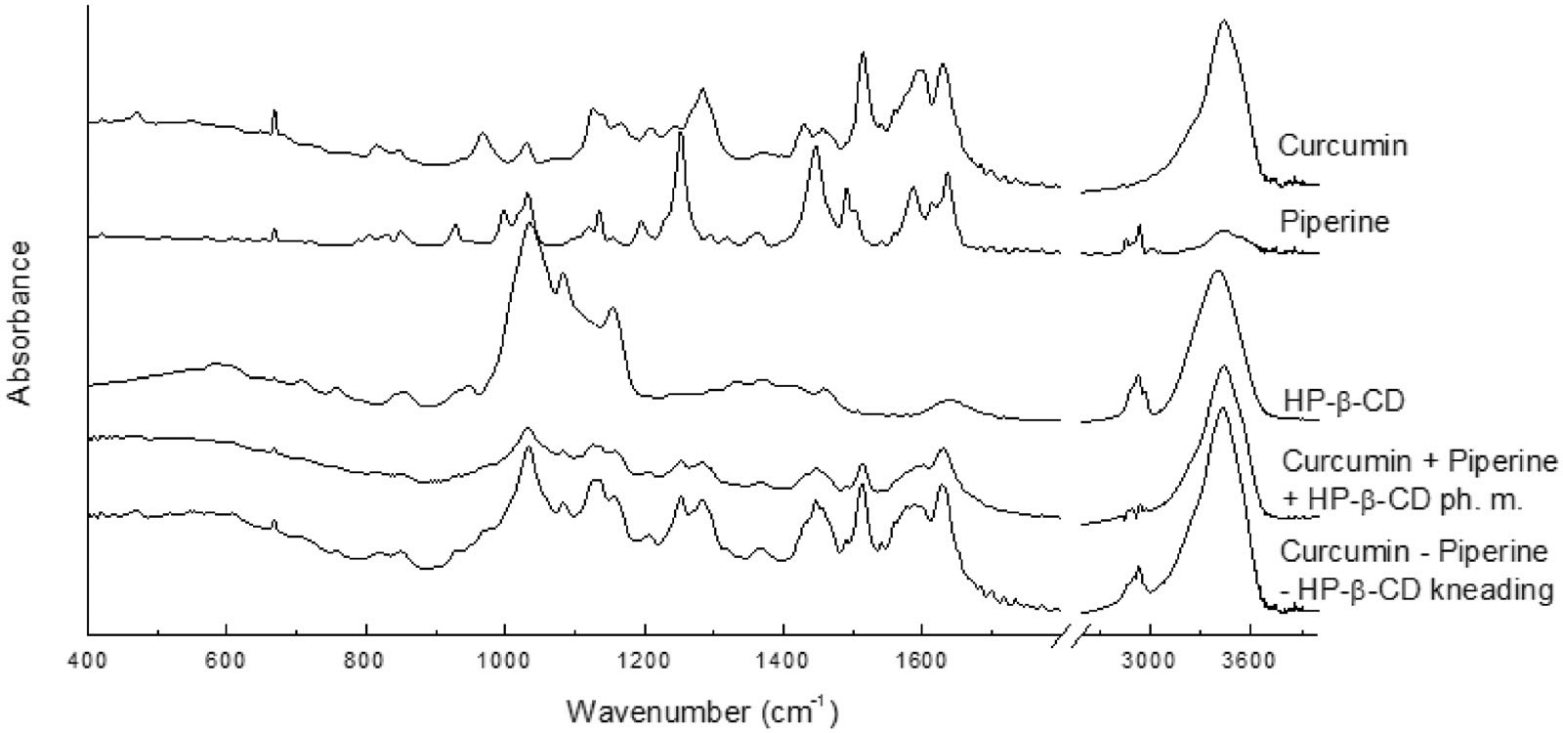
The experimental FT-IR of curcumin, piperine, their physical mixture, and system with hydroxypropyl-β-cyclodextrin (HP-β-CD) obtained by kneading method.

FT-IR spectroscopy was a technique that accurately indicated which curcumin and piperine domains were involved in the interaction with each other and the 2-hydroxypropyl-β-cyclodextrin carrier. The analysis of changes in the location and intensity of the characteristic bands was carried out based on the location of the characteristic bands identified as a result of comparison with the theoretical spectra of curcumin and piperine. Theoretical spectra were obtained by calculations using the DFT approach with a B3LYP functional and 6–31 G(d,p) basis set (Supplementary Materials Figures S1 and S2, Tables S1 and S2). The FT-IR spectra of curcumin, piperine, 2-hydroxypropyl-β-cyclodextrin their physical mixture, as well as their nutraceutical system are shown in the Figure 3. We observed the shift of the bands and changes in the shapes of bands and their intensities for the nutraceutical curcumin – piperine – 2-hydroxypropyl-β-cyclodextrin system. The changes are observed for both stretching vibrations of C–C, C=C, C–O, C=O bonds and bands corresponding to rocking, wagging and twisting vibration of the C–H bonds. For example, for the nutraceutical system of curcumin, piperine and cyclodextrin, the bands related to the C=O, and C=C bonds are shifted by 1–4 cm^−1^ and are observed at 1628, 1589 and 1576 cm^−1^. Similarly, some bands mainly associated with the stretching vibrations of the C–C, C–O bonds in piperine are shifted by a few cm^−1^, and are located at 1489, 1368, 1252, 1157 and 1032 cm^−1^. Slight shifts are also observed for bands related to the stretching vibration of the C–N bond in piperine and they are observed at 1138 and 1446 cm^−1^. The band observed at 3440 and 3433 cm^−1^ for curcumin and piperine, respectively and related to the stretching vibration of the O–H bond for the nutraceutical curcumin – piperine – 2-hydroxypropyl-β-cyclodextrin system is shifted to 3432 cm^−1^. In addition to the band maxima shifts, changes in band intensities are observed. The band at about 1589 cm^−1^ related to the stretching vibration of the C=C bonds in phenyl group and methyloendioxyphenyl rings has less intensity compared to ones in pure components. Despite the complexity of the nutraceutical system, it is possible to suggest that curcumin and piperine interact with each other. It is possible to suggest that phenyl rings are incorporated into the cavity of 2-hydroxypropyl-β-cyclodextrin and induce changes of physicochemical properties.

**Figure 4.**
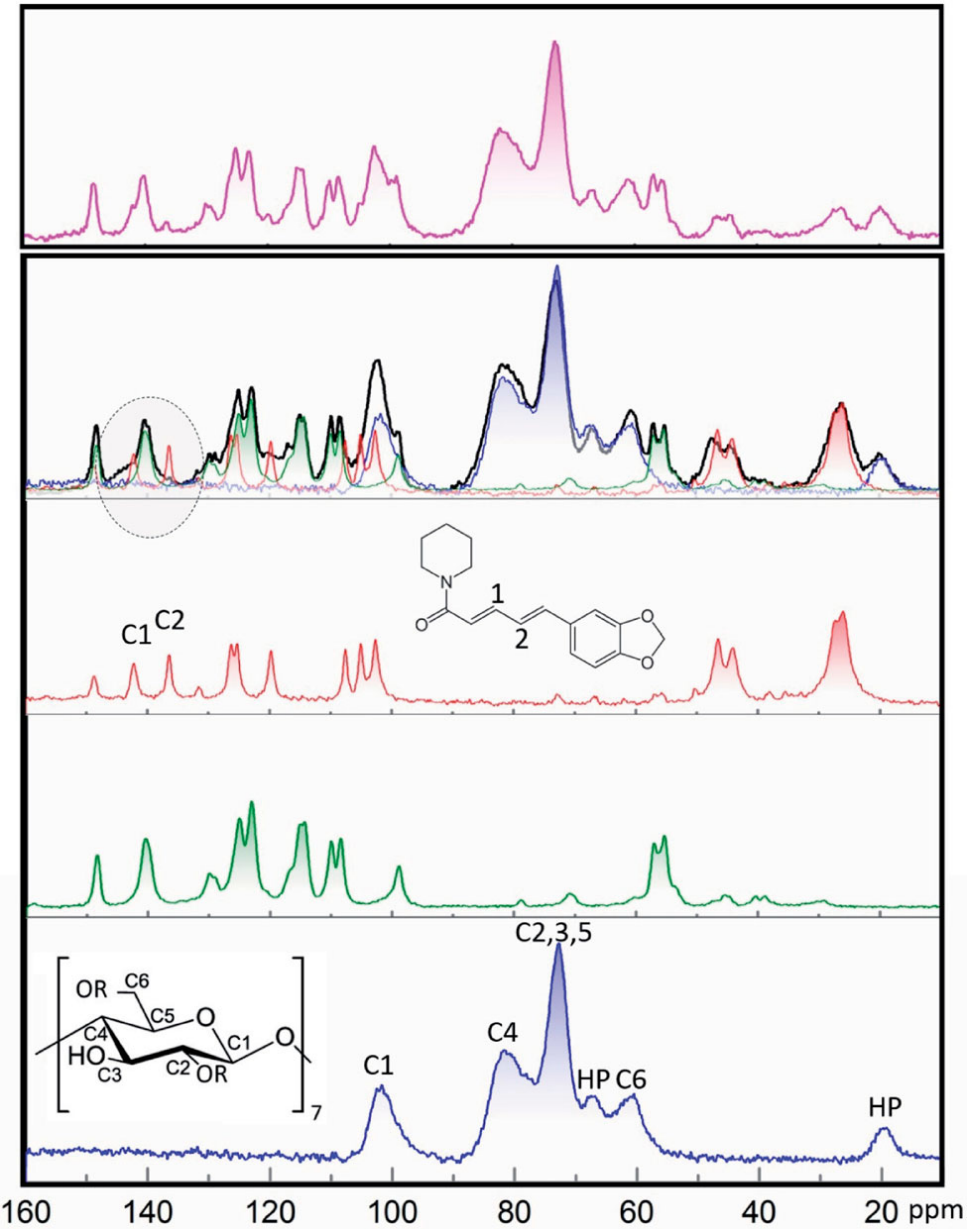
CP MAS ^13^C NMR spectra of HP-β-CD (blue), curcumin (green), piperine (red), kneaded compounds (black), and physically mixed compounds (magenta).

All three bulk components HP-β-CD^36^, piperine^37^, and curcumin (Supplementary Materials Figure S3) display characteristic ^13^C NMR signals positions as shown in Figure 4. The spectrum of physical mixture reflects simple superposition of spectra observed for individual components. On the contrary, it is worth pointing out that the spectrum recorded for kneaded mixture could not be reproduced by a simple addition of constituent NMR spectra (blue, green and red) taking into account solely the specified fractions of these three components. The spectrum of kneaded material reveals substantial changes observed in the case of signals corresponded to piperine. These signals seem undergo broadening upon kneading which is seen in particular for C1 and C2 signals highlighted by a dashed circle. Such broadening may indicate both stiffening of the molecule and increase of the chemical shifts distribution. Accordingly, directly form NMR data one can postulate quite significant piperine interaction with other components in this particular case.

**Figure 5.**
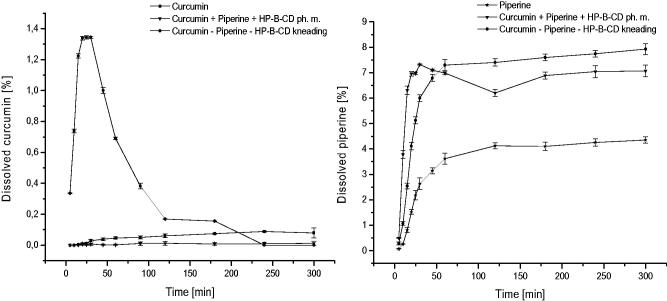
Apparent solubility of curcumin (A) and piperine (B) in phosphate buffer (pH 6.8).

The effect of interactions between nutraceutical system components: the curcumin and the piperine and their interactions with the 2-hydroxypropyl-β-cyclodextrin carrier was evaluated in relation to changes in solubility and permeability through membranes simulating gastrointestinal walls and the blood–brain barrier. As mentioned, the low solubility of curcumin is a limitation for its biological activity. Depending on the sources, curcumin is classified as belonging to group BCS II (poor solubility, high permeability) or group BCS IV (poor solubility, low permeability)^38–41^. Thus, according to the BCS classification, modification of curcumin solubility can induce changes in membrane permeability and potentially increase its activity *in vivo*. A similar situation is present in the case of piperine, which is also classified into group II BCS^42^. The combination of both curcumin and piperine with cyclodextrin increased their solubility (Figure 5). Curcumin showed an almost 48-fold increase in solubility, and it was the largest at 30 min from the start of solubility studies. There was no increase in curcumin solubility for the ternary physical mixture. The increase in piperine solubility was twofold greater. This increase is sufficient for the metabolic modifier function of piperine. According to f_1_ values, the dissolution profiles of curcumin and piperine in the nutraceutical system are significantly different than profiles for pure components.

**Figure 6.**
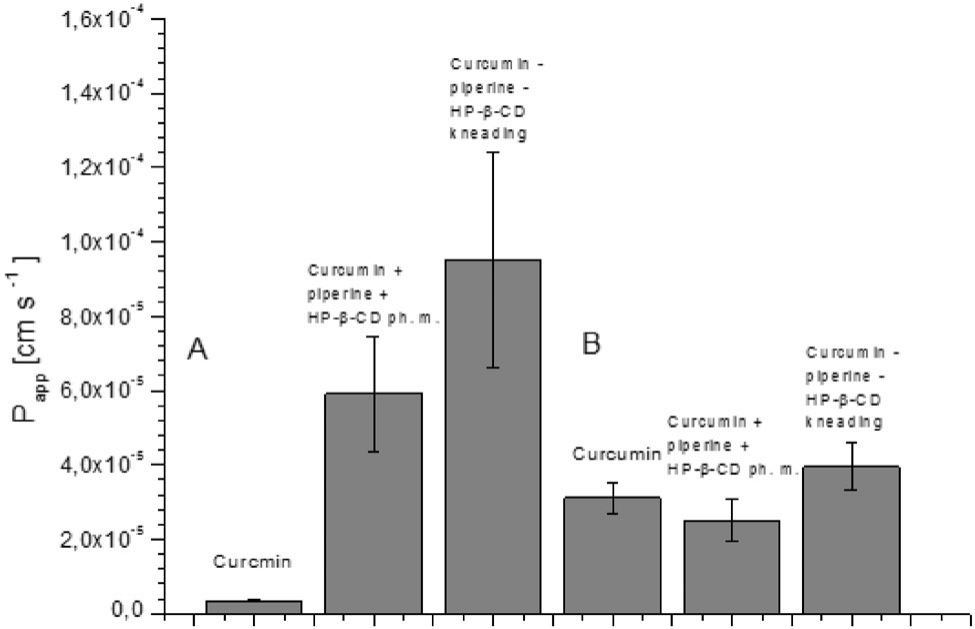
Values of apparent permeability coefficients of curcumin determined for gastrointestinal permeability (A) and permeability through the blood–brain barrier (B).

**Figure 7.**
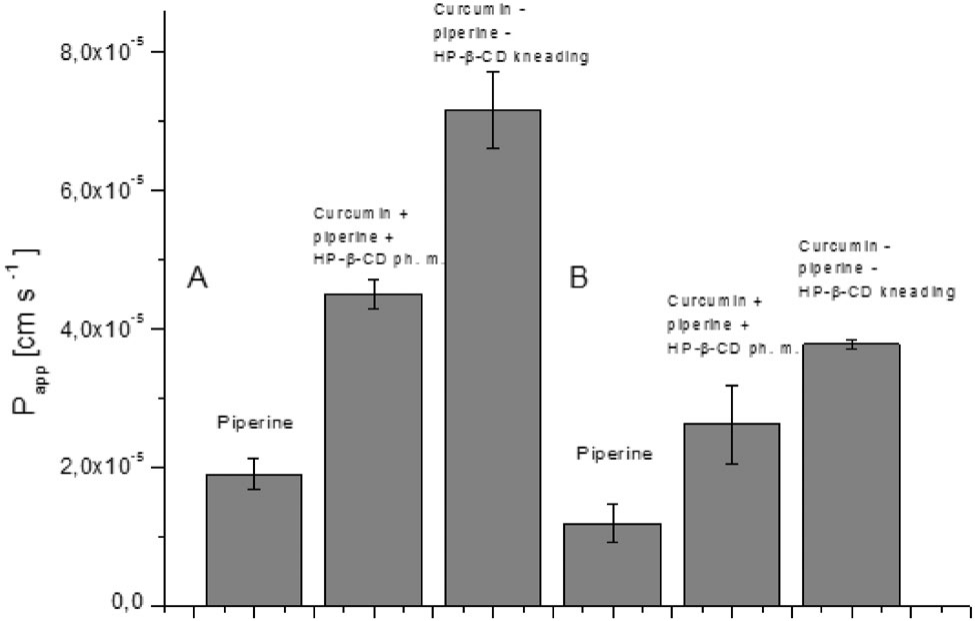
Values of apparent permeability coefficients of piperine determined for gastrointestinal permeability (A) and permeability through barrier blood–brain (B).

The consequence of curcumin and piperine solubility modification was an increase in their permeability through biological membranes simulating the walls of the gastrointestinal tract and the blood–brain barrier (Figures 6 and 7). Curcumin permeability studies using the GIT PAMPA model showed that it reaches *P_app_* values 3.46 × 10^−6 ^cm s^−1^ which allows it to be classified as a well permeable substance in accordance with the guidelines that *P_app_* value above 1 × 10^−6 ^cm s^−1^ describes substances as highly permeable^27^. Therefore, as a starting point, we assume that curcumin from the system under study belongs to the BCS classification group II. The higher permeability of curcumin in our research may be the result of the impact of other curcuminoids present in the raw material. Curcumin, after inclusion into the nutraceutical curcumin – piperine – 2-hydroxypropyl-β-cyclodextrin system, increased its permeability almost thirty times (*P_app_* 9.52 × 10^−5 ^cm s^−1^). It is worth noting that the preparation of the physical mixture of curcumin with piperine and 2-hydroxypropyl-β-cyclodextrin resulted in an increase in curcumin permeability (*P_app_* 5.91 × 10^−5 ^cm s^−1^). In the case of piperine, an increase in permeability was also determined. Similar to curcumin, the preparation of the physical mixture of curcumin, piperine and hydroxypropyl-β-cyclodextrin already resulted in higher permeability of piperine through GIT PAMPA membranes (*P_app_* 1.9 × 10^−5 ^cm s^−1^ *vs P_app_* 4.5 × 10^−5 ^cm s^−1^). The highest *P_app_* value for piperine was obtained on the nutraceutical curcumin – piperine – 2-hydroxypropyl-β-cyclodextrin system, and it was 7.15 × 10^−5 ^cm s^−1^. Lower *P_app_* values for piperine compared to curcumin correlate with the obtained absolute values of their solubility. Shao, B. et al.^43^ also confirmed that preparation of a self-emulsifying drug delivery of piperine increased its intestinal permeability about 1.3 time.

Curcumin and piperine have been shown to be able to act within the CNS^44^^,^^45^. As a pleiotropic effect of curcumin within the central nervous system, anti-inflammatory, antioxidant, and anti-protein-aggregate action are mentioned^46^. Curcumin’s profile of activity places it as an important nutraceutical candidate in the prevention of neurodegenerative diseases such as Alzheimer’s, Parkinson’s and stroke^47^^,^^48^. Neuroprotective effects have also been reported in piperine. S. Hua pointed out in preclinical studies on the effect of piperine on neural apoptosis in the case of brain damage in animals^49^. P. Shrivastava et al.^50^ confirmed that the anti-inflammatory effect of piperine might inhibit the development of Parkinson’s diseases. Our results confirm the possibility of curcumin (*P_app_* 3.11 × 10^−5 ^cm s^−1^) and piperine (1.19 × 10^−5 ^cm s^−1^) permeability through the blood–brain barrier. Both compounds were classified as well permeable because they achieved higher *P_app_* values than 4.0 × 10^−6 ^cm s^−1^ ^28^. The preparation of the curcumin and the piperine nutraceutical system with 2-hydroxypropyl-β-cyclodextrin increased their permeability through the blood–brain barrier (*P_app_* 3.95 × 10^−5 ^cm s^−1^ and 3.77 × 10^−5 ^cm s^−1^, respectively).

Antioxidant and radical scavenging properties of curcumin and piperine as well as raw materials, in which they are present: *Curcuma longa* and *Piper nigrum* are known^51–54^. In our studies, the antioxidant properties of the nutraceutical system of curcumin – piperine – 2-hydroxypropyl-β-cyclodextrin were tested using DPPH radical. Vitamin C was used as the reference with IC_50_ value 0.066 mg/mL (the concentration that is necessary to inhibit DPPH radical formation by 50% – calculated using linear regression analysis). IC_50_ value for curcumin is 0.161 mg/mL, while for piperine is 38.945 mg/mL. Changes in IC_50_ value (for curcumin to 0.151 mg/mL and for piperine to 0.110 mg/mL) suggest a beneficial effect of the nutraceutical curcumin – piperine – 2-hydroxypropyl-β-cyclodextrin system on the antioxidant properties of its components.

Curcumin’s anti-inflammatory effectiveness has been studied a number of times^55^^,^^56^. It is believed that it can be expressed by inhibiting the activity of many particles such as phospholipase, lipoxygenase, cyclooxygenase-II, thromboxane, prostaglandins, collagenase, elastase, hyaluronidase, interferon, tumour necrosis factor, interleukin-12^57^^,^^58^. In our research, we used a model of anti-inflammatory activity expressed in inhibition of hyaluronidase, conducting the turbidimetric evaluation. The results are presented in the form of the IC_50_ value, i.e. the concentration that inhibits the enzyme in 50%. Free curcumin inhibits hyaluronidase at the IC_50_ level of 6.04 mg/mL Similarly, piperine acts, although this effect is weaker (IC_50_ 13.26 mg/mL). The preparation of curcumin (29.59 mg/mL) and piperine (21.74 mg/mL) nutraceutical system with 2-hydroxypropyl-β-cyclodextrin did not cause the complete disappearance of hyaluronidase inhibition. Still, it reduced the anti-inflammatory effect of curcumin and piperine by activating this signalling pathway.

Antimicrobial activity of the curcumin – piperine – 2-hydroxypropyl-β-cyclodextrin nutraceutical system was established using the diffusion technique. Values of inhibition zones (mm) were measured in order to express the activity of the nutraceutical system against Gram (+), Gram (−) bacteria and pathogenic fungi (Table 1). Curcumin and piperine activities were used as reference values for inhibition zones (mm). In the case of the nutraceutical system of curcumin, piperine and 2-hydroxypropyl-β-cyclodextrin, an increase in activity against the following bacteria *C. butyricum, L. monocytogenes, B. subtillis, S. aureus, S. pyrogenes, P. aereuginosa* was reported. The enlargement of the inhibition zones (mm) is due to the synergy of curcumin and piperine but also the presence of 2-hydroxypropyl-β-cyclodextrin. There was a decrease in antimicrobial activity against *E. faecalis* and *E. coli*. Antimicrobial activity for curcumin and piperine in free forms was previously confirmed^59^^,^^60^. Also, 2-hydroxypropyl-β-cyclodextrin showed some antibacterial effect^61^, but more importantly, it increases bactericidal effect of other substances^62^^,^^63^. Cyclodextrins, according to many mechanisms, can counteract infections by decreasing antibiotic resistance via complexation^64^, lowering the cell-to-cell (quorum sensing) communication^65^, and blocking pore-forming toxins^66^.

**Table 1.** Antimicrobial activity of curcumin, piperine and curcumin – piperine – 2-hydroxypropyl-β-cyclodextrin system in the concentration 25 mg/mL when dissolved in methanol and filtered.

Microorganism	Inhibition zone (mm) – Agar well diffusion technique			
	Curcumin	Piperine	Curcumin + Piperine + HP-β-CD (ph. m.)	Curcumin – Piperine – HP-β-CD (kneading)
	25 mg/mL	25 mg/mL	25 mg/mL	25 mg/mL
*C. difficile* ATCC 9689	6.5	3.5	3.5	5.5
*C. butyricum* ATTC 860	5.5	5.5	4.0	7.0
*L. monocytogenes* ATCC 7644	5.0	5.0	4.5	6.0
*B. subtillis* ATCC 238557	8.5	7.0	6.0	10.0
*E. faecalis* ATTC 29212	10.5	9.0	2.0	4.5
*S. aureus* ATCC 25923	4.0	3.0	3.5	4.5
*S. pyrogenes* ATCC 19615	5.0	2.5	5.0	8.5
*E. coli* ATCC 25922	3.0	2.0	3.0	1.0
*K. pneumoniae* ATCC 31488	2.5	2.0	2.5	2.0
*P. mirabilis* ATCC 12453	3.0	1.0	3.0	2.0
*S. typhimurium* ATCC 14028	5.5	1.5	3.5	2.0
*P. aereuginosa* ATCC 27853	2.5	1.5	2.0	3.0
*E. aerogenes* ATCC 13048	2.5	1.0	1.0	1.0
*C. krusei* ATCC 14243	2.0	2.5	2.5	2.5
*C. albicans* ATTC 10231	4.5	2.5	2.5	3.0

**Figure 8.**
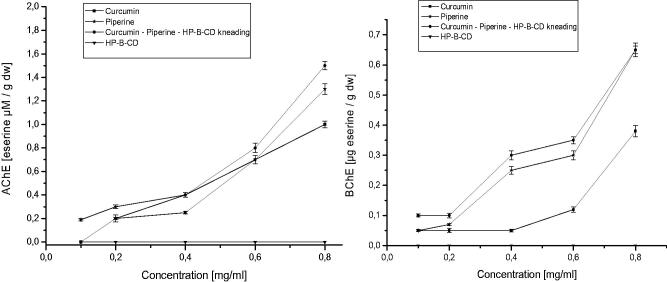
Activity of the nutraceutical system of curcumin, piperine and 2-hydroxypropyl-β-cyclodextrin vs curcumin vs piperine as acetylcholinesterase (A) and as butyrylcholinesterase inhibitors (B).

It has been proven that acetylcholine activity was significantly increased in Parkinson’s disease^67^. A. J. Akinyemi et al.^68^ demonstrated that curcumin altered acetylcholinesterase genes. Piperine also has an effect on acetylcholinesterase activity as an inhibitor^69^. What’s more, piperine strengthened the protective potential of curcumin against chronic unpredictable stress-induced cognitive impairment and oxidative damage in mice^70^. In our research, we evaluated the possibility acetylcholinesterase and butyrylcholinesterase inhibition by the nutraceutical curcumin – piperine – 2-hydroxypropyl-β-cyclodextrin system, with respect to enzyme inhibition by both components separately. Analysis of the results shows that curcumin and piperine after inclusion into 2-hydroxypropyl-β-cyclodextrin show stronger inhibition of acetylcholinesterase and butyrylcholinesterase. The nutraceutical curcumin – piperine – 2-hydroxypropyl-β-cyclodextrin system is more potent as an acetylcholinesterase inhibitor than butyrylcholinesterase (Figure 8). The ability to inhibit acetylcholinesterase and butyrylcholinesterase by the nutraceutical system is of particular importance in light of the increased blood–brain barrier permeability proven in our studies.

## 4. Conclusions

Using a simple and cheap preparation technique, we received a nutraceutical curcumin – piperine – 2-hydroxypropyl-β-cyclodextrin system. An increase in the solubility of curcumin and piperine, as well as permeability through membranes simulating the gastrointestinal walls and blood–brain barrier, suggest higher biological effectiveness *in vivo* of the nutraceutical system. *In vitro* screening of pharmacological activity shown synergy of curcumin and piperine concerning antioxidant and antibacterial activity as well as acetylcholinesterase and butyrylcholinesterase inhibition. Bearing in mind that piperine acts as a metabolic modifier of curcumin, we are justified in the hope that the use of the ternary nutraceutical system of curcumin, piperine and 2-hydroxypropyl-β-cyclodextrin will contribute to more effective prevention of diseases of infectious or neurodegenerative aetiology.

## Supplementary Material

Supplemental Material
