# Supplementary material for: Hydroxypropyl-β-cyclodextrin as an effective carrier of curcumin – piperine nutraceutical system with improved enzyme inhibition properties
Source: J Enzyme Inhib Med Chem. 2020 Sep 23;35(1):1811–21. doi: 10.1080/14756366.2020.1801670 (PMC7534320; doi:10.1080/14756366.2020.1801670)
Supplement: Supplemental Material [file IENZ_A_1801670_SM5659.pdf]

Supplementary Materials

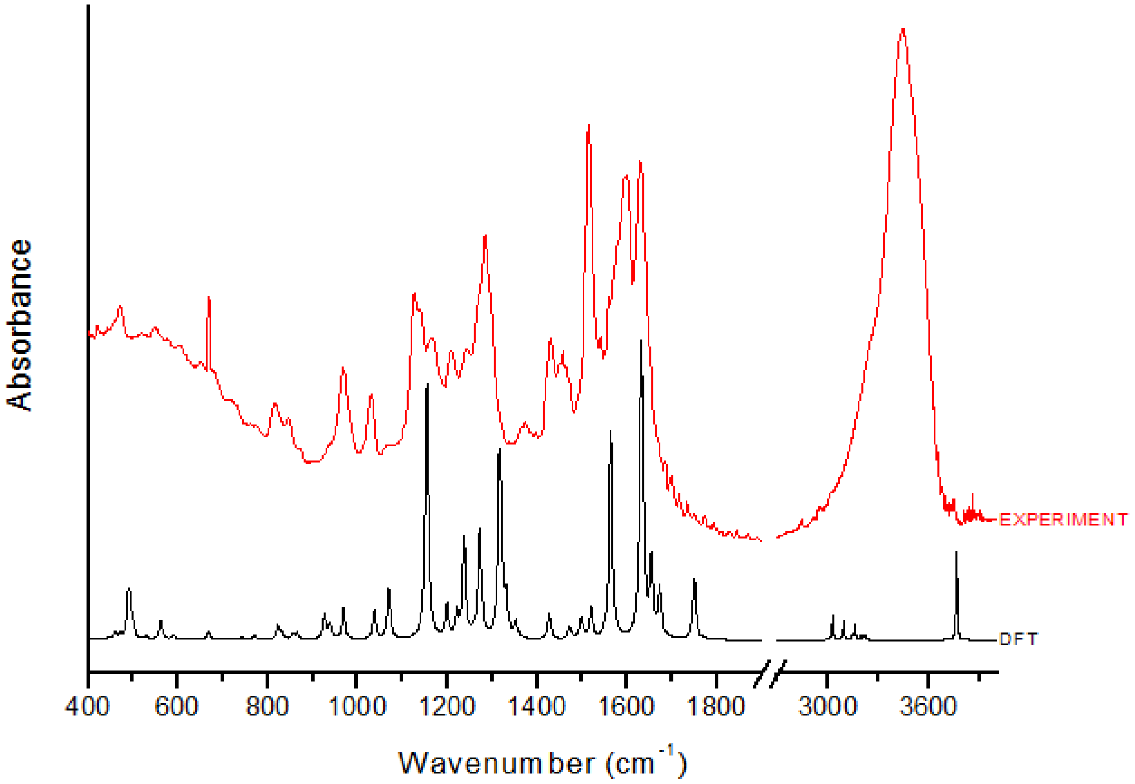

Figure S1. Calculation (black-DFT) and experimental (red) IR absorption spectra of curcumin at room temperature

Table S1. Selected characteristic vibronic features of curcumin theory with application of 6-31G(d,p) basis and experiment bands of curcumin. s-stretching, b-bending, w-wagging, r-rocking, t-twisting, oop-outside of the plane, def. - deformation.

Calculation (cm^{-1})	Experimental (cm^{-1})	Band assignment
561	470	Def. all molecule
582	548	Def. all molecule
823	815	Def. benzene rings
855	846	C-H w
865	872	C-H w
968	967	Def. all molecule
1038	1030	C-H w
1069	1064	CH_2 t + C-O s + def. benzene rings
1154	1126	C-C s
1198	1166	C-H w
1238	1208	C-O-H b + C-H r
1270	1243	C-O s + C-O-H b + C-H r + CH_2 t
1320	1283	C-O s + C-H r

1426	1373	C-O-H b + C-C s in benzene rings
1470	1429	C-H r
1502	1457	C-H w in CH_3
1519	1465	C-H r in CH_3
1564	1514	C-O s + C-C s
1635	1560	C-C s + C-O-H b + C-H r
1655	1577	C=C s + C-C s + C-O-H b
1674	1597	C=C s
1749	1628	C=O s + C=C s
3025	2841	C-H s in CH_3
3156	2941	C-H s
3760	3440	O-H s

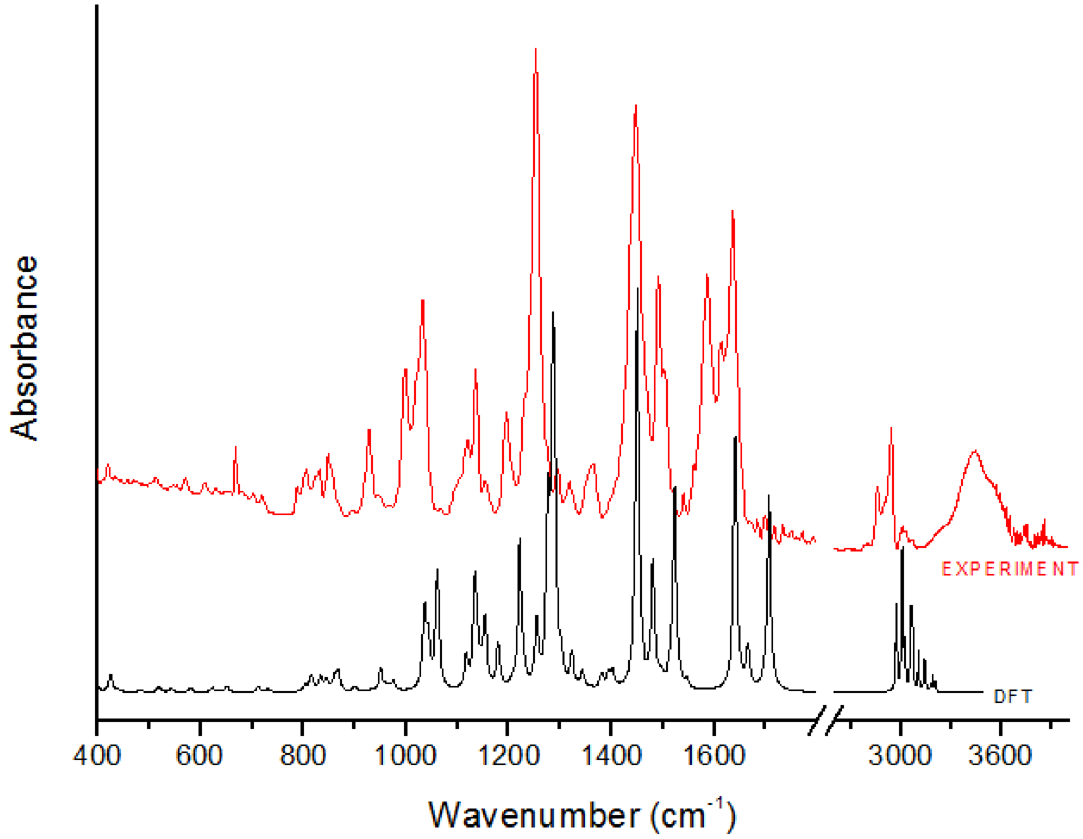

Fig. S2. Calculation (black-DFT) and experimental (red) IR absorption spectra of piperine at room temperature.

Table S2. Selected characteristic vibronic features of piperine theory with application of 6-31G(d,p) basis and experiment bands of piperine. s-stretching, b-bending, w-wagging, r-rocking, t-twisting, oop-outside of the plane, def. - deformation.

Calculation (cm^{-1})	Experimental (cm^{-1})	Band assignment
817	789	Def. piperidine ring
833	805	Breathing methyloendioxyphenyl ring
844	830	C-H w in piperidine ring

867	849	C-H w in methyloendioxyphenyl ring
950	928	C-O s asym.
1035	999	Def. piperidine ring
1061	1031	C-O s
1117	1099	C-O s sym. + C-H r in methyloendioxyphenyl ring
1134	1118	C-N s + C-H r
1154	1135	C-N s + C-H_2 t in piperidine ring
1179	1154	C-C s + C-H_2 t
1221	1197	C-H r
1254	1230	C-N s + C-H r
1286	1255	C-C s in methyloendioxyphenyl ring + C-O s + C-H r
1323	1296	C-H r
1342	1320	C-H r
1394	1363	C-C s in methyloendioxyphenyl ring
1450	1448	C-N s
1480	1464	C-C s in methyloendioxyphenyl ring + CH_2 t
1522	1491	C-O s + C-C s in methyloendioxyphenyl ring
1547	1540	CH_2 t in methyloendioxyphenyl ring
1640	1584	C=C s + C=O s
1666	1614	C=C s
1706	1636	C=O s
2973	2854	C-H s in piperidine ring
3104	2936	C-H s in methyloendioxyphenyl ring
3206	3016	C-H s

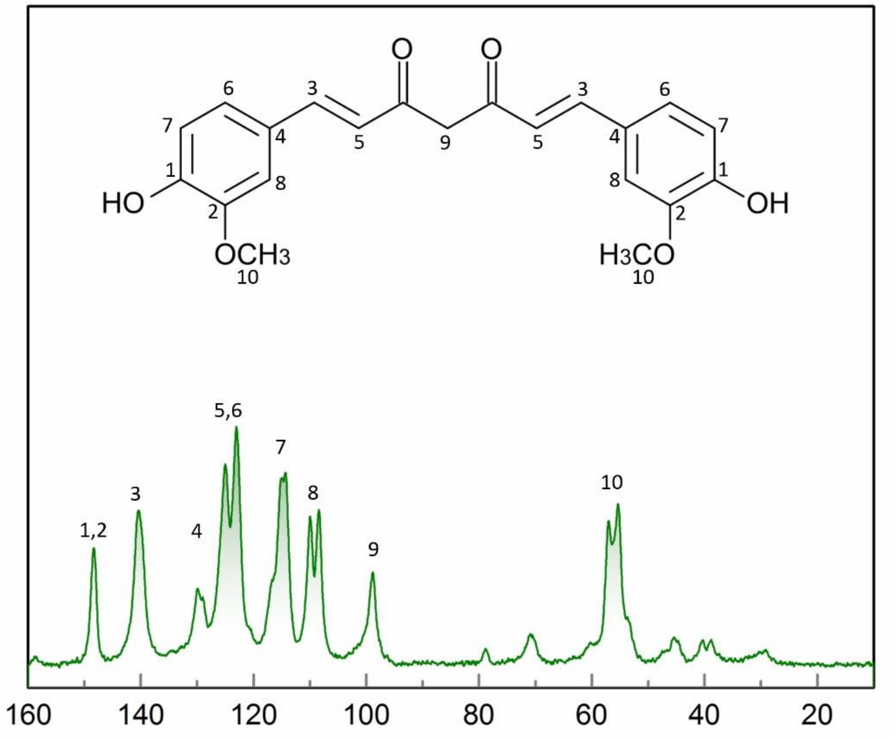

Fig. S3. ^{13}C NMR spectrum of curcumin.
